# RETRACTION: A Meta‐Analysis Showing the Quantitative Evidence Base of Perineural Nalbuphine for Wound Pain From Upper‐Limb Orthopaedic Trauma Surgery

**DOI:** 10.1111/iwj.70552

**Published:** 2025-04-18

## Abstract

**RETRACTION:** ShiW. , DongJ. , ChenJ.‐F. , and YuH. , “A Meta‐Analysis Showing the Quantitative Evidence Base of Perineural Nalbuphine for Wound Pain From Upper‐Limb Orthopaedic Trauma Surgery,” International Wound Journal 20, no. 5 (2023): 1476–1490, 10.1111/iwj.14002.36330591 PMC10088860

The above article, published online on 03 November 2022, in Wiley Online Library (http://onlinelibrary.wiley.com/), has been retracted by agreement between the journal Editor in Chief, Professor Keith Harding; and John Wiley & Sons Ltd. Following an investigation by the publisher, all parties have concluded that this article was accepted solely on the basis of a compromised peer review process. The editors have therefore decided to retract the article. The authors did not indicate their agreement with the retraction.

**RETRACTION:** W. Shi , J. Dong , J.‐F. Chen , and H. Yu , “A Meta‐Analysis Showing the Quantitative Evidence Base of Perineural Nalbuphine for Wound Pain From Upper‐Limb Orthopaedic Trauma Surgery,” International Wound Journal 20, no. 5 (2023): 1476–1490, 10.1111/iwj.14002.36330591 PMC10088860

The above article, published online on 03 November 2022, in Wiley Online Library (http://onlinelibrary.wiley.com/), has been retracted by agreement between the journal Editor in Chief, Professor Keith Harding; and John Wiley & Sons Ltd. Following an investigation by the publisher, all parties have concluded that this article was accepted solely on the basis of a compromised peer review process. The editors have therefore decided to retract the article. The authors did not indicate their agreement with the retraction.

